# Global trends in men's and women's acceptance of intimate partner violence, 1999–2022: an analysis of population-based survey data from 83 countries

**DOI:** 10.1016/j.eclinm.2025.103199

**Published:** 2025-04-09

**Authors:** Irina Bergenfeld, Vince Nguyen, Katjana Wiederkehr, Alexandria R. Hadd, Eva Portillo Molina, Cari Jo Clark, Robin A. Richardson

**Affiliations:** a Hubert Department of Global Health, Emory University Rollins School of Public Health, Atlanta, GA, USA; b Department of Epidemiology, Emory University Rollins School of Public Health, Atlanta, GA, USA; c Department of Epidemiology, Columbia University Mailman School of Public Health, New York, NY, USA

**Keywords:** Intimate partner violence, Attitudes, Lower- and middle-income countries

## Abstract

**Background:**

Intimate partner violence (IPV) is a pervasive public health issue affecting women worldwide. Attitudes about the acceptability of IPV are correlated with IPV perpetration, experience, and help-seeking; therefore, monitoring trends in attitudes is an important way to track progress towards gender equality. This study presents the most comprehensive assessment of global trends in IPV acceptance to date.

**Methods:**

Using population-based surveys including 4.37 million women and 1.22 million men across 83 countries (1999–2022), we modeled average yearly changes in the percentage of women and men with permissive IPV attitudes at the country, world region, and global levels, as well as by age group (≤25 and > 25 years).

**Findings:**

Broad variation in the acceptability of IPV was observed in men's (range = 2%–85%) and women's attitudes (range = 2%–92%) across countries. Women tended to be more accepting than men at both earlier and more recent timepoints. On average, the percentage of individuals responding that IPV was acceptable in at least one circumstance decreased significantly over time for men (3.79% [−5.02%, −2.57%]/year) and women (6.80% [−7.83%, −5.77%]/year), with no substantive differences by age group.

**Interpretation:**

The acceptability of IPV has declined substantially in the last 20 years, especially among women. Heterogeneity in changes in IPV-related attitudes across countries suggests that pooled estimates should be interpreted cautiously and that national or subnational trends may be more informative. Future research should investigate specific country- and local-level factors that may be driving changes in IPV-supportive attitudes.

**Funding:**

Eunice Kennedy Shriver National Institute of Child Health and Human Development (5R00HD104896).

**Research in context**

**Evidence before this study**

Intimate partner violence (IPV) is a global threat to women's health and a key development indicator for gender equality. Men's, women's, and community attitudes about the acceptability of IPV have been consistently described as key correlates of IPV experience, perpetration, and help-seeking in cross-sectional research and are often measured as outcomes in IPV reduction interventions. We searched Google Scholar, PubMed, SCOPUS, PsycINFO, and Web of Science, without language restrictions, using search terms relevant to IPV attitudes (e.g., IPV acceptance, IPV attitudes, IPV rejection, etc.) up until June 4, 2024. We also searched reference lists of relevant studies to widen our search. We found four multi-country studies linking attitudes and IPV perpetration, victimization, and/or help-seeking. We found three multi-country studies examining changes in IPV attitudes: one focused on women in African countries up to 2013, one focused on men in lower- and middle-income countries up to 2022, and one included men's and women's attitudes up to 2009. The latter study found that in most countries, the proportion of women who held permissive attitudes about IPV against wives decreased over two time points in most of the included 26 studies, with similar patterns observed in the smaller sample of 15 countries with men's data. The single-gender studies found similar patterning, with decreases in the acceptability of IPV observed overall.

**Added value of this study**

The continued pervasiveness of IPV worldwide warrants an updated analysis of global trends in IPV-related attitudes to include both men and women and to include more than two timepoints of data collection, where available. Using data from 83 countries and a sample of 4.37 million women and 1.22 million men, we have estimated annual percentage-point changes in men's and women's attitudes at the country, World Bank region, and global levels. We also conducted stratified analyses by age (≤25 and > 25 years). We harmonized population-based data across 473 surveys to achieve the most comprehensive global analysis to date.

**Implications of all the available evidence**

We found that in almost all countries in the sample, the proportion of permissive attitudes toward IPV was greater among women that men in both earlier and later surveys, but that women's IPV acceptance was declining at a faster rate than men's. Both men's and women's acceptance of IPV declined substantively in pooled global models and across World Bank regions, except for men's attitudes in South Asia, which remained stable. We found no notable differences in age groups at the global level, although some differences emerged at the region and country level. This study confirms that trends of declining acceptance observed in the first decade of the 2000s have continued. However, lack of evidence of similar progress toward the elimination of IPV suggests that the relationship between IPV and attitudes is far from straightforward and should be examined in future research. Moreover, a large degree of heterogeneity in pooled estimates suggests that country-level factors, including violent conflict and women's literacy, may be influencing both attitudes and IPV prevalence in complex ways.

## Introduction

Intimate partner violence (IPV) is defined as harmful behaviors perpetrated by a spouse, partner, or ex-partner. These behaviors include physical and sexual violence, as well as emotional and economic abuse and controlling behaviors.^1^ Globally, an estimated 27% of women and girls 15 and older have experienced physical and/or sexual IPV within their lifetimes, and 13% have experienced physical and/or sexual IPV within the past year.^1^ In recognition of the high social, health, and economic costs of IPV, the United Nations has made reducing the proportion of women and girls subjected to IPV a key indicator of Sustainable Development Goal 5, Gender Equality.^2^ In parallel, the past two decades have seen increased monitoring, advocacy, and prevention efforts by national, international, and community stakeholders.^3^

IPV-related attitudes, whether men's, women's, or community members', are considered important correlates of women's IPV experience and of men's perpetration^4^, ^5^, ^6^, ^7^, ^8^, ^9^ and are often measured as outcomes in IPV prevention interventions.^10^ For example, social norms change approaches, which aim to change behavior by shifting harmful social rules about the acceptability of IPV, often aggregate individual attitudinal measures to assess their impact.^4^ IPV acceptance is also a key determinant of help-seeking among individuals experiencing IPV,^6^ which may be discouraged by social norms. Accordingly, monitoring changes in acceptance over time can provide important information about progress towards reducing violence against women, providing appropriate care for survivors, and promoting gender equity.

Early evidence from the first decade of the 2000s suggests that acceptance of IPV decreased in many countries during this time. A 26-country study using population-based surveys found that women in almost all countries sampled became less accepting toward IPV.^3^ Although data was sparser for men's attitudes, similar patterns were observed in a sample of 15 countries.^3^ The study concluded that the decrease in acceptance of IPV was not primarily explained by demographic shifts, but was rather observed within cohorts, which the author attributed to the dissemination of anti-violence against women messages by international actors.^3^ More recent analyses, including one that found IPV-accepting attitudes among women in 30 African countries decreased on average by almost 2% per year between 1999 and 2013,^11^ and a DHS report showing declining men's acceptance of IPV in 18 of 22 LMICs,^12^ suggest that declines in the acceptability of physical IPV have continued.

Gender differences in the acceptability of IPV vary across low- and middle-income countries (LMICs), with one large multi-country study in 49 countries finding greater overall acceptance among women^6^ and another study with 72 lower-, middle, and higher-income countries finding greater acceptance among men.^13^ A smaller 2016 study using men's and women's data from 13 LMICs found that in countries where overall acceptance of IPV was low, men tend to be more accepting, but where overall acceptance is high, women tend to be more accepting.^14^ Differences by region have also been observed, with women tending to have higher IPV acceptance than men in Sub-Saharan Africa and South/Southeast Asia, and men tending to have higher acceptance in Europe, Central Asia, Latin America, and the Caribbean.^6^

Interestingly, recent work modeling changes in reported IPV over the past 20 years found almost no change in overall IPV prevalence worldwide, and mixed results when considering physical, sexual, and emotional IPV separately.^11^^,^^15^ The lack of strong evidence that IPV against women has decreased within the past two decades despite notable shifts in women's IPV acceptance in the early 2000s warrants further investigation: has progress on attitudes stalled within the past decade and a half, or have trends in men and women's attitudes begun to diverge from each other? The current study investigates trends in IPV acceptance by gender and age group, examining changes at the country, region, and global levels.

## Methods

### Data sources and sample

We use harmonized, population-based, nationally representative survey data collected between the years 1999 and 2022 to provide the most comprehensive assessment to date on national and world region trends in men's and women's attitudes about the acceptability of IPV. Data used in this analysis was collected by the Demographic and Health Survey (DHS) and Multiple Indicator Cluster Survey (MICS) programs, which have tracked attitudes about violence against women for over two decades in LMICs using the same standard items.^16^ Both the DHS and MICS are population-based surveys that use multistage sampling to achieve nationally representative samples of women and men aged 15–49 through repeated cross-sectional surveys.^16^ (Note: Some countries define eligible age ranges differently from the standard DHS/MICS sample, expanding the men's sample to 54 or 59 years or excluding individuals under 18.) Rich data sources such as DHS and MICS are useful because their questions remain the same across countries and over time, allowing for accurate estimation of national trends in IPV-related attitudes. The DHS and MICS ask both men and women about their attitudes on the acceptability of IPV, presenting an opportunity to compare trends by gender. To model changes over time, we included all countries that collected information on men's and/or women's attitudes about IPV at a minimum of two timepoints.

### Measures

Structured questions within the MICS and DHS ask a respondent whether a husband is justified in beating his wife if she (1) goes out without telling him, (2) neglects the children, (3) argues with him, (4) refuses to have sex with him, or (5) burns the food. For each question, response options include “agree”, “disagree”, or “don't know”. We recoded “don't know” responses (<2%) to missing and calculated the prevalence of permissive attitudes toward IPV based on the proportion of individuals who selected “agree” to at least one of the five items separately for men and women.

### Analysis

We aggregated responses to the year and country level using sampling weights provided by DHS and MICS, thus providing prevalence estimates that are representative of men and women aged 15–49 at the country level. For surveys administered over more than one year, we collapsed information into one year, using the year with a larger sample size, due to concerns of uneven rollout of surveys in a single calendar year (e.g., oversampling of urban adults). We estimated the average yearly change in the percentage of men and women who felt that IPV against wives was justified in at least one circumstance in each country using generalized linear models with a log link. We calculated pooled estimates for men and women at the global level, and because we hypothesized that regional differences in trends would emerge, we also pooled estimates at the World Bank region level for regions with >2 countries. We calculated pooled estimates using random effects models due to evidence of high heterogeneity in effect estimates across countries.^17^ We tested for differences by age group, estimating separate models restricted to men and women ≤25 years old and >25 years old. Because individuals under 25 and under are at the highest risk of experiencing recent IPV,^1^ we hypothesized that attitudes might be changing more rapidly in younger people and that demographic shifts could be partially responsible for overall changes in IPV acceptance. We also visualized average yearly changes by country for men and women using a heat map generated in Microsoft Excel. Finally, we graphed point prevalences for men's and women's IPV-related attitudes in each country over time to visualize trends in each of these outcomes in STATA. Data cleaning and management was performed in STATA, while all models were run in R.

### Ethics committee approval

This study was determined to be exempt from IRB oversight by the Institutional Review Board of Emory University. Interviewers from DHS and MICS surveys obtained verbal informed consent from all participants prior to data collection.

### Role of the funding source

The funders of the study had no role in study design, data collection, data analysis, data interpretation, or writing of the report.

## Results

The final sample included women's IPV-related attitudes data from 4,373,336 women from 83 countries, and men's IPV attitudes from 1,224,739 men from 57 countries. The sample included data from 473 distinct surveys and represented countries from six of the seven World Bank regions: South Asia (n = 6), East Asia and Pacific (n = 8), Europe and Central Asia (n = 15), Latin America and Caribbean (n = 12), Middle East and North Africa (n = 4), and Sub-Saharan Africa (n = 38). The North America region was not represented as it consists exclusively of high-income countries, for which data is not available.

The proportion of individuals with permissive attitudes toward IPV ranged widely by country and year, from 1.58% (Serbia, 2019) to 92.23% (Guinea, 2012) for women and 1.92% (Cuba, 2019) to 84.64% (Timor-Leste, 2009) for men (Supplemental Material, Table S1). Interestingly, individuals over 25 years old were less likely to find IPV acceptable; for men, this figure was 28.81% for older men versus 37.01% for younger men. The difference in women's opinions was less pronounced, with 37.34% of women over 25 and 40.55% of women 25 and under reporting permissive IPV attitudes.

**Fig. 1:**
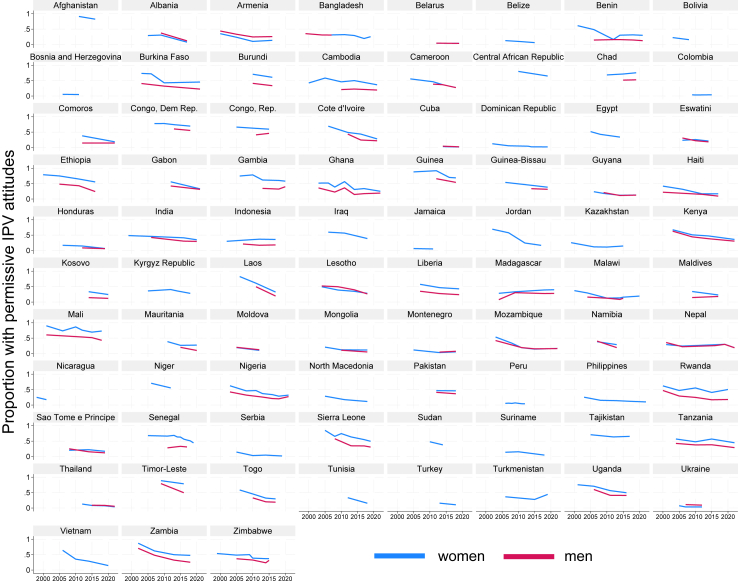
**Proportion of women and men with permissive attitudes about IPV by country (n = 83), 1999–2022**.

In most countries, women's IPV acceptance remained higher than men's over time (Fig. 1). Only two countries (Armenia and Albania) among those with data for men and women during the same time period (n = 56), showed men's acceptance as consistently greater than women's across all years measured. In five countries (Belarus, Cuba, Guyana, Nepal, and Thailand), men's and women's acceptance remained roughly equal over time. The overall directionality of trends in men's and women's attitudes was roughly the same within countries, with a few exceptions: Senegal, Comoros, Republic of Congo, and the Maldives. In these four countries, women's acceptance of IPV decreased, while men's increased, although women's remained higher at the most recent timepoint. The most common pattern was declines in men and women's permissive IPV attitudes at roughly the same rate but with women's acceptance higher overall, seen in 12 countries. Converging patterns, where women's acceptance decreased more quickly than men's, was observed in nine countries, while three countries showed men's acceptance decreasing faster than women's (Timor-Leste, Rwanda, and Mauritania). The only country with increases in both men's and women's acceptance was Madagascar. While many of the countries with the sharpest declines in IPV acceptance reported high levels of acceptance in earlier surveys (>50%), this was not universally the case.

**Fig. 2:**
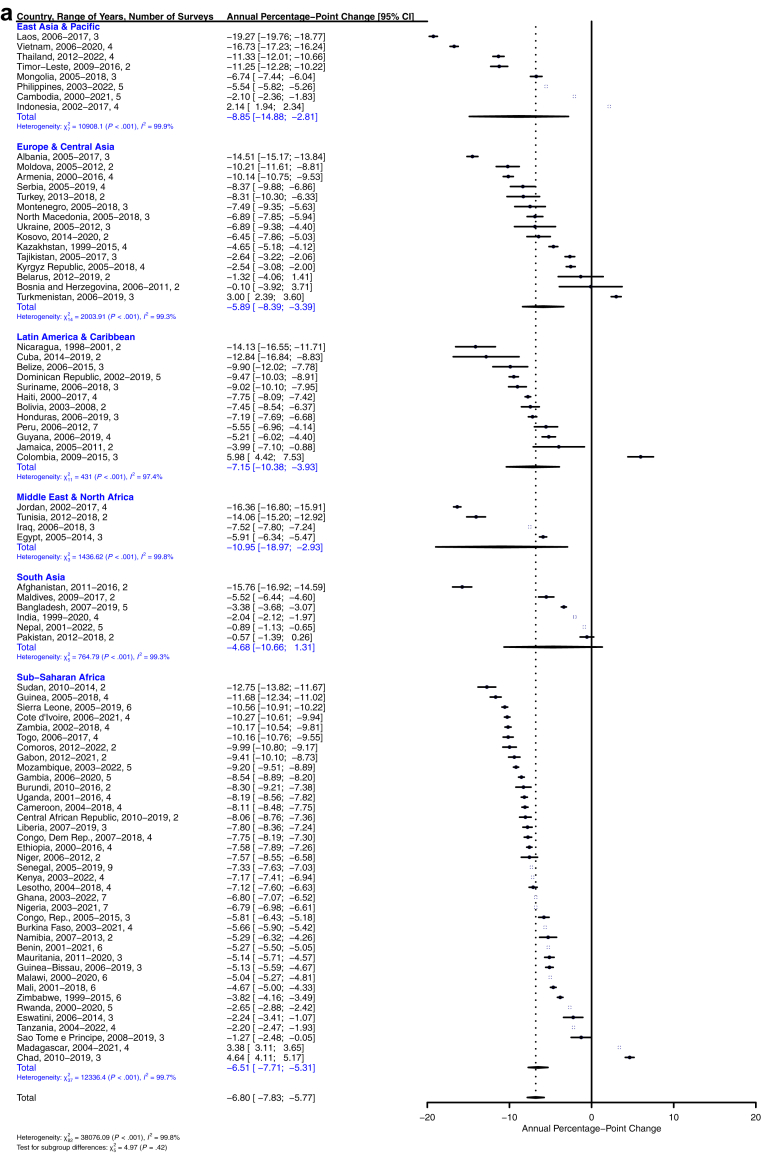

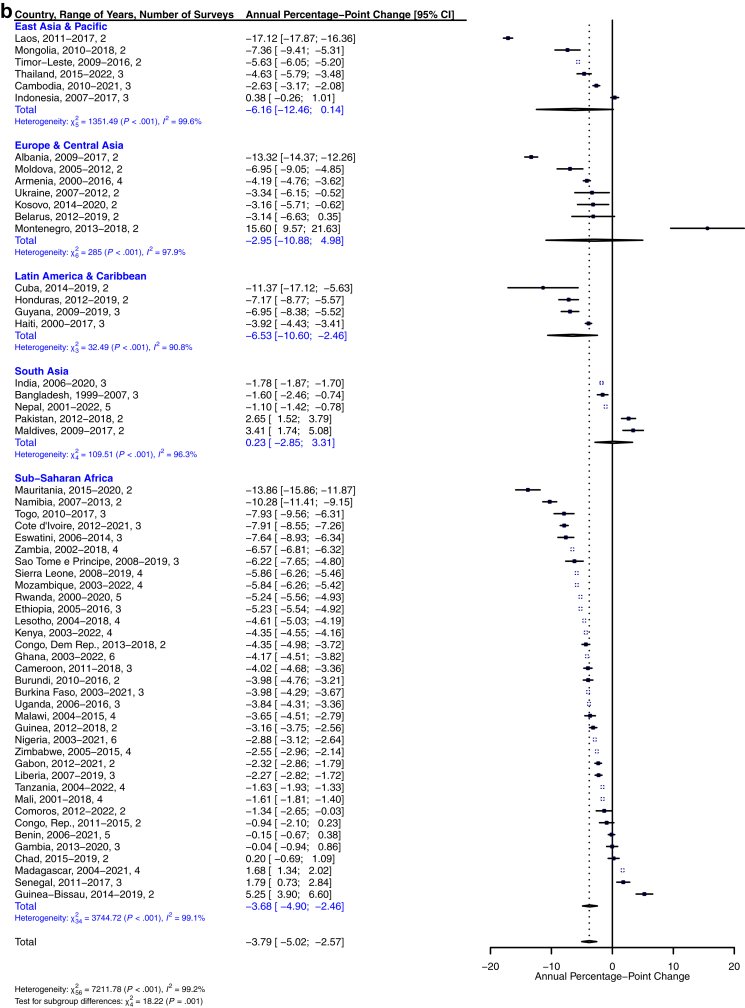
**a. Global, regional, and country-level annual percentage-point changes in women's permissive IPV attitudes across 83 countries. b. Global, regional, and country-level annual percentage-point changes in men's permissive IPV attitudes across 57 countries**.

**Fig. 3:**
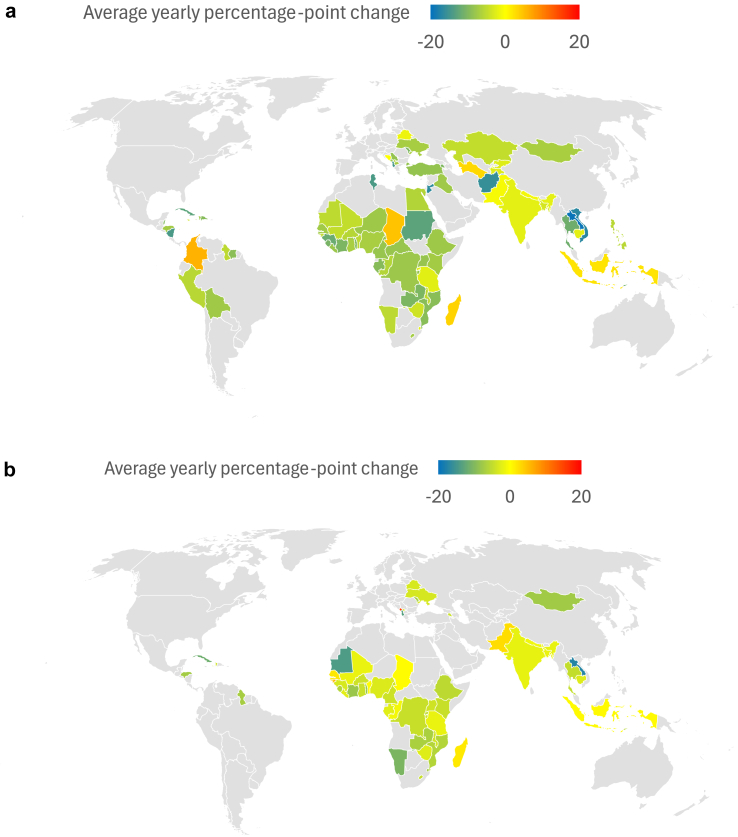
**a. Average yearly percentage-point change in women's permissive IPV attitudes by country. b. Average yearly percentage-point change in men's permissive IPV attitudes by country**.

Women's acceptance of IPV decreased significantly in pooled analyses, at an average of −6.80% [−7.83%, −5.77%] per year across the 83 countries in the sample (Figs. 2a and 3a). Additionally, IPV-accepting attitudes declined by at least 4% annually in all six World Bank regions. The prevalence of accepting attitudes toward physical IPV increased in only five of the 83 countries in the sample: Chad, Indonesia, Madagascar, Turkmenistan, and Colombia. IPV acceptance remained stable (<1% change) in Pakistan, Nepal, and Bosnia and Herzegovina. In Laos, Vietnam, Jordan, and Afghanistan, acceptance of IPV decreased by 15% per year or more, on average. In the age-stratified models, the rate of change for women 25 years and under was −6.83% [−7.85%, −5.82%], while that of women over 25 years was −6.86% [−7.96%, −5.76%], suggesting negligible differences by age (Supplemental Material Figures S1 & S2). While average annual changes were also broadly similar at the regional level across age groups, a few notable differences emerged at the country level. For example, acceptance of IPV increased for older women in Bosnia and Herzegovina while decreasing among younger women; the reverse was true in Jamaica.

Men's attitudes toward IPV followed similar trends, declining by −3.79% [−5.02%, −2.57%] annually (Figs. 2b and 3b). Of these, the East Asia & Pacific and Latin America & Caribbean regions showed the largest pooled decreases in men's attitudes toward IPV (−6.16% and −6.53%, respectively), although the majority of countries in other regions also showed significant decreases (Fig. 3b). Madagascar, Senegal, Guinea-Bissau, Pakistan, Maldives, and Montenegro were the only countries across the 57 in the sample with significant increases in the proportion of men who accepted IPV, with Indonesia, Republic of Congo, Benin, Gambia, and Chad remaining unchanged. Laos, Mauritania, and Albania showed the largest decreases in IPV acceptance among men. While the rate of change in men 25 years and under (−3.96% [−5.33%, −2.60%]) was greater than that of older men (−3.76% [−4.99%, −2.52%]), this difference was not substantive (Supplemental Material, Figures S3 & S4). At the regional level, only Latin America and the Caribbean showed a large degree of difference in estimates for younger (−9.00% [−17.32, −0.67] per year) versus older men (−4.36% [−5.89, −2.83] per year). Differences between older and younger men at the country level were in degree of change rather than in the direction of change, with several notable differences in the magnitude of trend estimates in younger versus older men. For example, in Montenegro, younger men's IPV acceptance increased by about 21% annually compared to older men's 11% annually, and in Cuba, young men's acceptance decreased by about 19% annually compared to older men's 9%. Exceptions included Kosovo, where younger men's IPV acceptance decreased by about 6% per year and older men's remained unchanged, and the Maldives, where older men's IPV acceptance remained stable while younger men's increased by about 11% annually.

## Discussion

We found that the prevalence of men and women reporting permissive attitudes toward IPV generally decreased between 1999 and 2022, reflecting a continuation of trends observed in the first decade of the 2000s.^11^ Overall, women reported more acceptance of IPV, perhaps reflecting prior literature observing a greater tendency for women to accept IPV in LMICs^13^ and an overrepresentation of Sub-Saharan Africa in the sample, a region where women's IPV acceptance tends to be higher than men's.^6^ Women's acceptance of IPV declined at almost double the rate of men's acceptance overall. While younger individuals of both genders were more accepting of IPV, the rate of attitudinal change in older and younger groups was essentially the same.

While these significant and consistent shifts in attitudes are encouraging, a lack of evidence in the current literature^11^^,^^15^ supporting similar declines in IPV prevalence suggests that the relationship between these factors is far from straightforward.^18^, ^19^, ^20^ In comparing our attitudes data with a recent analysis of trends in women's IPV experience using DHS data,^15^ all of the countries where IPV acceptance increased in our study have seen decreases in physical IPV during the same timespan; conversely, countries where physical IPV has been increasing have observed decreases in IPV acceptance. Moreover, many of the countries with large decreases in physical IPV have shown very modest declines in IPV acceptance.^15^ IPV-related attitudes are often described as predictors of IPV experience and/or perpetration, yet much of the evidence for this relationship comes from cross-sectional data, highlighting the need for more rigorous longitudinal studies to tease out the intricacies of this relationship and its interaction with gender. Because witnessing IPV in the household and community is also associated with IPV acceptance,^9^ IPV prevalence may also be driving attitudes in a bi-directional relationship. Efforts to reduce IPV may also encourage individuals to respond in socially desirable ways that do not necessarily accurately reflect their attitudes, creating a disconnect between reported attitudes and actual norms. Finally, the decreasing acceptability of IPV may actually increase women's willingness to report overall,^6^ further complicating the relationship between attitudes and reported IPV prevalence. This could partially explain why IPV seems to be increasing in some countries where attitudes are improving, and conversely, why IPV seems to be decreasing in some countries where IPV acceptability is increasing.

Indeed, researchers have noted large gaps in attitudes, perceived norms, and actual behavior in relation to IPV, even at the individual level,^21^ with perceived community norms having a larger influence on men's perpetration than individual attitudes.^18^ Therefore, social norms change approaches, including mass-media campaigns^22^ and community mobilization,^23^ may be most appropriate in countries where men's IPV attitudes have become less favorable or where progress has stagnated in comparison to women's, as in much of South Asia. In particular, multilevel interventions such as the education-entertainment program *Soul City IV*, which aim to shift attitudes around IPV while promoting action from community bystanders, law enforcement, and government^10^ may be necessary to strengthen the link between changes in attitudes and changes in behavior. At the country level, stronger democratic institutions are associated with lower IPV acceptance among men, but not among women.^6^ Thus, changes in national governance over the past two decades may partially account for trends in men's attitudes.

In many of the LMICs in our sample, women's IPV acceptance remained higher than men's in recent surveys, despite more rapid declines. One of the strongest correlates of women's, but not men's, IPV acceptance, is female literacy.^6^ Likewise, the economic status of women is associated with decreased acceptance of IPV among both men and women, but more strongly for women.^5^^,^^24^ Large-scale improvements in education for women in LMICs in the past few decades^25^ may partially explain the large declines in women's IPV acceptance and divergent attitudes by age and gender in some countries. While this suggests that efforts to expand educational and economic opportunities for women and girls are a promising means to improve attitudes around IPV, these efforts may not translate to reductions in IPV without access to services and norms change around help-seeking to allow women to leave abusive relationships.^23^^,^^26^

Women are especially vulnerable to IPV in settings affected by violent conflict,^27^ which is also associated with higher IPV acceptance among both men and women in cross-sectional studies.^6^ Although IPV acceptance in many countries that have experienced armed conflicts or other civil unrest in the past three decades, including Timor-Leste, Sierra Leone, and Afghanistan, remains high, we found notable shifts toward more favorable IPV attitudes in the years following those conflicts. These findings suggest that in settings where acceptance of IPV has become elevated in the wake of exogenous shocks, IPV-related attitudes can return to lower levels over the long term.^20^^,^^23^ Since conflict has larger impacts on men's IPV attitudes, public health responses to conflict must consider its gendered impacts to avoid reversals in progress toward gender equality.

In a 2013 analysis of changes in IPV attitudes, Pierotti theorized that anti-violence against women “scripts” promulgated by international health and development organizations were driving increasing rejection of IPV among women globally.^3^ There is now mounting evidence that exposure to mass media (e.g., radio, television, and social media), whether generally,^3^ or specifically designed to change norms and attitudes surrounding violence against women,^3^^,^^22^^,^^28^ may be a key strategy for effecting large-scale decreases in IPV acceptability among both men and women. One advantage of such strategies is that they may be implemented at the national level or be targeted to specific communities and populations within a country. The current study highlights key differences by gender and age at the country level that may be further explored to tailor media strategies for reducing violence against women, especially in countries where attitudes are diverging among men and women or among older versus younger populations.

We did not find evidence of broad homogeneity in the direction of IPV attitudes at the global or regional levels. Likewise, examples of countries where IPV acceptance increased were present across all regions. The substantial heterogeneity we observed in country-level changes in IPV-related attitudes indicates that considering country-specific and subnational factors, including history of conflict, strength of democratic institutions, and women's access to education and economic opportunities, may be more informative than examining broader regional and world-wide trends. Future research should investigate national and subnational influences that may be driving changes in both attitudes and IPV to better tailor interventions to the local context. This will be particularly useful in countries where notable differences exist across age groups and by gender, such as Senegal, the Maldives, and Jamaica.

The large sample size, inclusion of men's and women's data, and use of harmonized measures are notable strengths of this analysis. However, some limitations warrant consideration. Firstly, although DHS/MICS questionnaires use the same standardized language, there is some variation in actual question wording across countries and administrations. These differences, which include variations in both the preamble and the wording of specific items, have been shown to account for a significant portion of variation in responses to these questions across countries.^7^^,^^29^ Moreover, the DHS and MICS are translated into multiple languages, even within the same country, possibly resulting in slightly different translations of the same items. Although formal testing has found the attitude items to be comparable across countries within the Latin America & Caribbean region,^30^ it has not been established whether these items are equivalent within other regions or over time within the same country due to differences in survey administration. It must also be noted that the attitudes questions used in the DHS/MICS use dichotomous responses rather than Likert scales, which might offer more nuanced information. Furthermore, the questions only ask about the acceptability of a relatively severe form of physical IPV to the exclusion of other forms of IPV. Secondly, 31 of 140 country-level analyses only had two surveys available, meaning that limited information was used to detect changes over time. Moreover, only four countries comprised the Middle East & North Africa region, calling into question the representativeness of pooled regional estimates. Finally, DHS and MICS are administered only in LMICs, and our findings should not be generalized to high-income countries. Data may not be available for countries and years in which civil unrest occurred, which limits the analysis to periods when countries were stable and committed to administering large-scale health surveys.

The world is currently not on track to achieve Sustainable Development Goal 5.2., the elimination of violence against women and girls. While substantial decreases in the acceptability of IPV globally are encouraging, IPV against women remains highly prevalent. Further examination of the complex relationship between IPV and IPV-related attitudes will require more rigorous, longitudinal research. In addition, examination of high-level contextual drivers of both IPV and its acceptability, including local laws and policies, women's education, protective legislation, and violent conflict, is needed to develop appropriate, effective, and targeted prevention and response efforts to address this key women's health issue.

## Contributors

IB, VN, and AH directly accessed the data. IB and AH validated the data. All authors made the decision to submit, and IB submitted the manuscript.

**IB:** Formal analysis; Writing- original draft; Writing-review & editing; Data curation; Visualization; Software.

**VN:** Software; Writing-review & editing; Data curation; Visualization; Formal Analysis.

**KW:** Writing-review & editing; Data curation.

**AH:** Writing-review & editing; Data curation; Software.

**EM:** Writing-review & editing; Data curation.

**CC:** Writing-review & editing.

**RAR:** Writing-review & editing; Conceptualization; Funding Acquisition; Supervision; Methodology; Project administration.

## Data sharing statement

All data underlying this study is available from the Demographic and Health Survey Program at https://dhsprogram.com and Multiple Indicator Cluster Survey at https://mics.unicef.org.

## Declaration of interests

The authors declare no conflict of interest.
