# Supplementary material for: Global trends in men's and women's acceptance of intimate partner violence, 1999–2022: an analysis of population-based survey data from 83 countries
Source: eClinicalMedicine. 2025 Apr 9;83:103199. doi: 10.1016/j.eclinm.2025.103199 (PMC12008140; doi:10.1016/j.eclinm.2025.103199)

**Supplemental Material for Global trends in men’s and women’s acceptance of intimate partner violence, 1999 – 2022: An analysis of population-based survey data from 83 countries**

Table S1………………………………………………………..…………………………………………………………….pg 2

Figure S1….………………………………………………………………………………………………………….…….pg 11

Figure S2……………………………………………….………………………………………………………….……….pg 12

Figure S3……………………………………………….……………………………………………………….………….pg 13

Figure S4……………………………………………….…………………………………………………….…………….pg 14

**Table S1. Percentage of women and men with permissive IPV attitudes by country and year**

|  | **Women** | **Men** |
| --- | --- | --- |
| **Afghanistan** |  |  |
| **2011** | 91.83 |  |
| **2016** | 82.66 |  |
| **Albania** |  |  |
| **2005** | 30.35 |  |
| **2009** | 30.12 | 36.49 |
| **2017** | 7.07 | 11.63 |
| **Armenia** |  |  |
| **2000** | 32.78 | 42.79 |
| **2005** | 22.63 | 30.96 |
| **2010** | 9.41 | 20.63 |
| **2016** | 10.18 | 22.83 |
| **Bangladesh** |  |  |
| **1999** |  | 36.16 |
| **2004** |  | 32.12 |
| **2007** | 31.02 | 31.9 |
| **2011** | 32.57 |  |
| **2014** | 28.31 |  |
| **2017** | 20.12 |  |
| **2019** | 25.47 |  |
| **Belarus** |  |  |
| **2012** | 4.06 | 5.01 |
| **2019** | 3.72 | 4.01 |
| **Belize** |  |  |
| **2006** | 12.3 |  |
| **2011** | 8.68 |  |
| **2015** | 5.22 |  |
| **Benin** |  |  |
| **2001** | 60.74 |  |
| **2006** | 46.98 | 13.64 |
| **2012** | 16.37 | 14.94 |
| **2014** | 36.03 | 16.61 |
| **2018** | 31.97 | 15.12 |
| **2021** | 29.47 | 13.31 |
| **Bolivia** |  |  |
| **2003** | 22.12 |  |
| **2008** | 16.16 |  |
| **Bosnia and Herzegovina** |  |  |
| **2006** | 4.78 |  |
| **2011** | 4.76 |  |
| **Burkina Faso** |  |  |
| **2003** | 71.64 | 45.4 |
| **2006** | 71.89 |  |
| **2010** | 43.52 | 33.41 |
| **2021** | 44.45 | 21.83 |
| **Burundi** |  |  |
| **2010** | 73.11 | 43.39 |
| **2016** | 61.79 | 34 |
| **Cambodia** |  |  |
| **2000** | 37.99 |  |
| **2005** | 56.34 |  |
| **2010** | 45.88 | 21.61 |
| **2014** | 50.74 | 26.08 |
| **2021** | 37.43 | 16.45 |
| **Cameroon** |  |  |
| **2004** | 55.85 |  |
| **2011** | 46.66 | 38.06 |
| **2014** | 36.15 | 38.55 |
| **2018** | 28.44 | 28.37 |
| **Central African Republic** |  |  |
| **2010** | 80.01 |  |
| **2019** | 65.26 |  |
| **Chad** |  |  |
| **2010** | 69.7 |  |
| **2015** | 75.82 | 50.85 |
| **2019** | 77.6 | 51.25 |
| **Colombia** |  |  |
| **2009** | 3.89 |  |
| **2010** | 2.14 |  |
| **2015** | 3.23 |  |
| **Comoros** |  |  |
| **2012** | 40.49 | 16.6 |
| **2022** | 19.2 | 14.51 |
| **Congo, Dem Rep.** |  |  |
| **2007** | 77.41 |  |
| **2010** | 77.27 |  |
| **2013** | 75.45 | 59.98 |
| **2018** | 61.75 | 48.02 |
| **Congo, Rep.** |  |  |
| **2005** | 68.32 |  |
| **2011** | 61.21 | 41.54 |
| **2015** | 54.34 | 40.01 |
| **Cote d'Ivoire** |  |  |
| **2006** | 64.87 |  |
| **2012** | 48.14 | 41.31 |
| **2016** | 41.12 | 23.55 |
| **2021** | 25.83 | 20.23 |
| **Cuba** |  |  |
| **2014** | 3.27 | 3.51 |
| **2019** | 1.67 | 1.92 |
| **Dominican Republic** |  |  |
| **2002** | 8.63 |  |
| **2007** | 3.94 |  |
| **2013** | 4.46 |  |
| **2014** | 2.01 |  |
| **2019** | 2.21 |  |
| **Egypt** |  |  |
| **2005** | 50.2 |  |
| **2008** | 39.44 |  |
| **2014** | 35.76 |  |
| **Eswatini** |  |  |
| **2006** | 23.23 | 31.3 |
| **2010** | 27.63 | 23.08 |
| **2014** | 19.88 | 16.28 |
| **Ethiopia** |  |  |
| **2000** | 84.96 |  |
| **2005** | 81.4 | 52.22 |
| **2011** | 68.64 | 44.78 |
| **2016** | 63.35 | 27.77 |
| **Gabon** |  |  |
| **2012** | 50.55 | 38.79 |
| **2021** | 29.58 | 31.39 |
| **Gambia** |  |  |
| **2006** | 74.31 |  |
| **2010** | 74.66 |  |
| **2013** | 58.55 | 32.49 |
| **2018** | 49.97 | 26.36 |
| **2020** | 51.47 | 34.03 |
| **Ghana** |  |  |
| **2003** | 48.77 | 32.1 |
| **2006** | 46.76 |  |
| **2008** | 36.7 | 21.3 |
| **2011** | 44.07 | 25.72 |
| **2014** | 28.34 | 12.5 |
| **2017** | 32.44 | 16.54 |
| **2022** | 19.31 | 15.24 |
| **Guinea** |  |  |
| **2005** | 87.67 |  |
| **2012** | 92.23 | 66.79 |
| **2016** | 70.88 |  |
| **2018** | 68.24 | 55.1 |
| **Guinea-Bissau** |  |  |
| **2006** | 53.25 |  |
| **2014** | 41.96 |  |
| **2019** | 36.54 |  |
| **Guyana** |  |  |
| **2006** | 18.1 |  |
| **2009** | 16.53 | 19.35 |
| **2014** | 10.2 | 9.69 |
| **2019** | 10.82 | 10.36 |
| **Haiti** |  |  |
| **2000** | 40.34 | 20.45 |
| **2006** | 31.51 |  |
| **2012** | 16.79 | 14.66 |
| **2017** | 16.59 | 9.97 |
| **Honduras** |  |  |
| **2006** | 15.57 |  |
| **2012** | 12.39 | 9.39 |
| **2019** | 6.21 | 5.58 |
| **India** |  |  |
| **1999** | 50.64 |  |
| **2006** | 47.49 | 41.93 |
| **2016** | 44.87 | 31.97 |
| **2020** | 38.32 | 33.79 |
| **Indonesia** |  |  |
| **2002** | 24.98 |  |
| **2007** | 31.01 | 16.34 |
| **2012** | 34.77 | 17.38 |
| **2017** | 32.18 | 17.01 |
| **Iraq** |  |  |
| **2006** | 59.55 |  |
| **2011** | 51.47 |  |
| **2018** | 36.83 |  |
| **Jamaica** |  |  |
| **2005** | 6.13 |  |
| **2011** | 4.86 |  |
| **Jordan** |  |  |
| **2002** | 65.43 |  |
| **2007** | 52.56 |  |
| **2012** | 22.61 |  |
| **2017** | 14.15 |  |
| **Kazakhstan** |  |  |
| **1999** | 30.49 |  |
| **2006** | 10.53 |  |
| **2010** | 12.4 |  |
| **2015** | 14.19 |  |
| **Kenya** |  |  |
| **2003** | 68.96 | 63.67 |
| **2009** | 53.18 | 43.84 |
| **2014** | 42.01 | 36.28 |
| **2022** | 32.43 | 27.25 |
| **Kosovo** |  |  |
| **2014** | 32.89 | 14.88 |
| **2020** | 24.73 | 12.27 |
| **Kyrgyz Republic** |  |  |
| **2005** | 37.67 |  |
| **2012** | 35.19 |  |
| **2014** | 32.94 |  |
| **2018** | 29.88 |  |
| **Laos** |  |  |
| **2006** | 82.37 |  |
| **2011** | 59.89 | 50.31 |
| **2017** | 29.84 | 16.31 |
| **Lesotho** |  |  |
| **2004** | 48.64 | 51.69 |
| **2009** | 37.16 | 47.96 |
| **2014** | 33.37 | 38.85 |
| **2018** | 23.58 | 22.74 |
| **Liberia** |  |  |
| **2007** | 61.49 | 30.98 |
| **2013** | 42.55 | 24.55 |
| **2019** | 37.3 | 24.01 |
| **Madagascar** |  |  |
| **2004** | 28.22 | 8.41 |
| **2009** | 32.58 | 29.27 |
| **2018** | 40.6 | 28.54 |
| **2021** | 40.71 | 27.84 |
| **Malawi** |  |  |
| **2000** | 36.05 |  |
| **2004** | 28.73 | 16.16 |
| **2010** | 12.61 | 12.64 |
| **2014** | 12.89 | 8.06 |
| **2015** | 16.37 | 12.73 |
| **2020** | 18.89 |  |
| **Maldives** |  |  |
| **2009** | 30.94 | 13.97 |
| **2017** | 22.14 | 18.26 |
| **Mali** |  |  |
| **2001** | 89.83 | 62.77 |
| **2006** | 76.8 |  |
| **2010** | 87.76 |  |
| **2012** | 77.04 | 53.43 |
| **2015** | 73.07 | 51.39 |
| **2018** | 79.73 | 46.31 |
| **Mauritania** |  |  |
| **2011** | 38.63 |  |
| **2015** | 25.19 | 20.34 |
| **2020** | 27.48 | 9.65 |
| **Moldova** |  |  |
| **2005** | 21.13 |  |
| **2012** | 11.2 |  |
| **Mongolia** |  |  |
| **2005** | 20.69 |  |
| **2010** | 10.11 | 8.9 |
| **2018** | 9.3 | 4.83 |
| **Montenegro** |  |  |
| **2005** | 11.05 |  |
| **2013** | 2.73 | 4.51 |
| **2018** | 6.12 | 9.3 |
| **Mozambique** |  |  |
| **2003** | 54.15 | 41.37 |
| **2008** | 35.86 |  |
| **2011** | 21.16 | 18.77 |
| **2015** | 13.86 | 16.61 |
| **2022** | 19.19 | 14.9 |
| **Namibia** |  |  |
| **2007** | 35.76 | 40.98 |
| **2013** | 28.66 | 21.38 |
| **Nepal** |  |  |
| **2001** | 28.77 | 34.11 |
| **2006** | 23.22 | 20.7 |
| **2016** | 28.55 | 22.93 |
| **2019** | 29.81 | 29.59 |
| **2022** | 18.55 | 17.16 |
| **Nicaragua** |  |  |
| **1998** | 24.37 |  |
| **2001** | 16.95 |  |
| **Niger** |  |  |
| **2006** | 70.92 |  |
| **2012** | 60.33 |  |
| **Nigeria** |  |  |
| **2003** | 65.21 | 43.3 |
| **2008** | 44.03 | 29.8 |
| **2011** | 46.24 |  |
| **2013** | 35.23 | 24.9 |
| **2016** | 34.05 | 21.65 |
| **2018** | 28.11 | 20.22 |
| **2021** | 29.34 | 23.89 |
| **North Macedonia** |  |  |
| **2005** | 21.49 |  |
| **2011** | 14.51 |  |
| **2018** | 9.97 |  |
| **Pakistan** |  |  |
| **2012** | 42.46 | 32.05 |
| **2018** | 41.63 | 37.5 |
| **Peru** |  |  |
| **2006** | 4.33 |  |
| **2007** | 5.22 |  |
| **2008** | 4.66 |  |
| **2009** | 5.75 |  |
| **2010** | 4.93 |  |
| **2011** | 3.61 |  |
| **2012** | 3.53 |  |
| **Philippines** |  |  |
| **2003** | 24.18 |  |
| **2008** | 14.2 |  |
| **2013** | 13.01 |  |
| **2017** | 10.95 |  |
| **2022** | 9.17 |  |
| **Rwanda** |  |  |
| **2000** | 63.81 | 48.06 |
| **2005** | 48.37 | 29.6 |
| **2010** | 56.25 | 24.76 |
| **2015** | 41.44 | 17.13 |
| **2020** | 49.61 | 17.26 |
| **Sao Tome e Principe** |  |  |
| **2008** | 19.91 | 21.78 |
| **2014** | 19.09 | 13.83 |
| **2019** | 17.76 | 11.3 |
| **Senegal** |  |  |
| **2005** | 65.89 |  |
| **2011** | 60.1 | 24.26 |
| **2013** | 64.27 |  |
| **2014** | 57.22 |  |
| **2015** | 56.54 | 24.72 |
| **2016** | 48.69 |  |
| **2017** | 45.85 | 27.04 |
| **2018** | 44.04 |  |
| **2019** | 39.17 |  |
| **Serbia** |  |  |
| **2005** | 6.29 |  |
| **2010** | 2.9 |  |
| **2014** | 3.85 |  |
| **2019** | 1.58 |  |
| **Sierra Leone** |  |  |
| **2005** | 85.2 |  |
| **2008** | 67.37 | 59.45 |
| **2010** | 74.26 |  |
| **2013** | 64.84 | 34.45 |
| **2017** | 53.11 | 32.8 |
| **2019** | 48.83 | 30.11 |
| **Sudan** |  |  |
| **2010** | 47.37 |  |
| **2014** | 34.28 |  |
| **Suriname** |  |  |
| **2006** | 13.31 |  |
| **2010** | 12.48 |  |
| **2018** | 4.73 |  |
| **Tajikistan** |  |  |
| **2005** | 75.07 |  |
| **2012** | 66.89 |  |
| **2017** | 68.15 |  |
| **Tanzania** |  |  |
| **2004** | 59.99 | 42.31 |
| **2010** | 53.91 | 42.55 |
| **2015** | 58.63 | 39.84 |
| **2022** | 48.64 | 31.86 |
| **Thailand** |  |  |
| **2012** | 13.12 |  |
| **2015** | 8.66 | 8.78 |
| **2019** | 7.24 | 8.51 |
| **2022** | 3.52 | 5.74 |
| **Timor-Leste** |  |  |
| **2009** | 89.54 | 84.64 |
| **2016** | 78.78 | 56.43 |
| **Togo** |  |  |
| **2006** | 53.31 |  |
| **2010** | 43.04 | 30.5 |
| **2014** | 28.86 | 17.65 |
| **2017** | 28.19 | 18.86 |
| **Tunisia** |  |  |
| **2012** | 30.34 |  |
| **2018** | 14.93 |  |
| **Turkey** |  |  |
| **2013** | 13.26 |  |
| **2018** | 9.01 |  |
| **Turkmenistan** |  |  |
| **2006** | 38.24 |  |
| **2015** | 26.78 |  |
| **2019** | 49.05 |  |
| **Uganda** |  |  |
| **2001** | 76.93 |  |
| **2006** | 70.61 | 59.41 |
| **2011** | 58.55 | 42.88 |
| **2016** | 49.09 | 40.17 |
| **Ukraine** |  |  |
| **2005** | 4.98 |  |
| **2007** | 3.62 | 11.37 |
| **2012** | 2.97 | 9.6 |
| **Vietnam** |  |  |
| **2006** | 64.27 |  |
| **2010** | 36.24 |  |
| **2014** | 28.34 |  |
| **2020** | 10.97 |  |
| **Zambia** |  |  |
| **2002** | 86.32 | 69.54 |
| **2007** | 62.8 | 48.38 |
| **2013** | 47.49 | 30.94 |
| **2018** | 45.79 | 24.24 |
| **Zimbabwe** |  |  |
| **1999** | 51.34 |  |
| **2005** | 47.88 | 36.39 |
| **2009** | 49.46 |  |
| **2010** | 39.66 | 33.11 |
| **2014** | 37.39 | 23.26 |
| **2015** | 38.73 | 32.37 |

**Figure S1. Global, regional, and country-level annual percentage-point changes in permissive IPV attitudes, women 25 years and under across 83 countries**


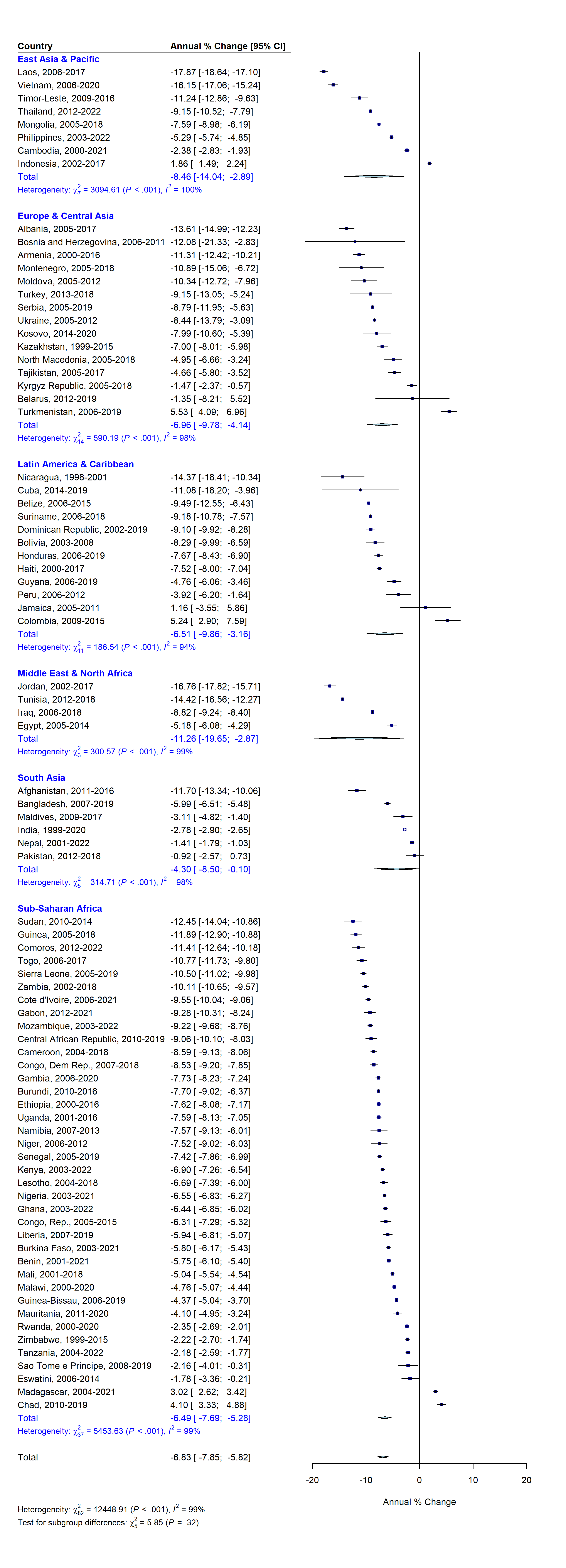


**Figure S2. Global, regional, and country-level annual percentage-point changes in permissive IPV attitudes, women over 25 years across 83 countries**


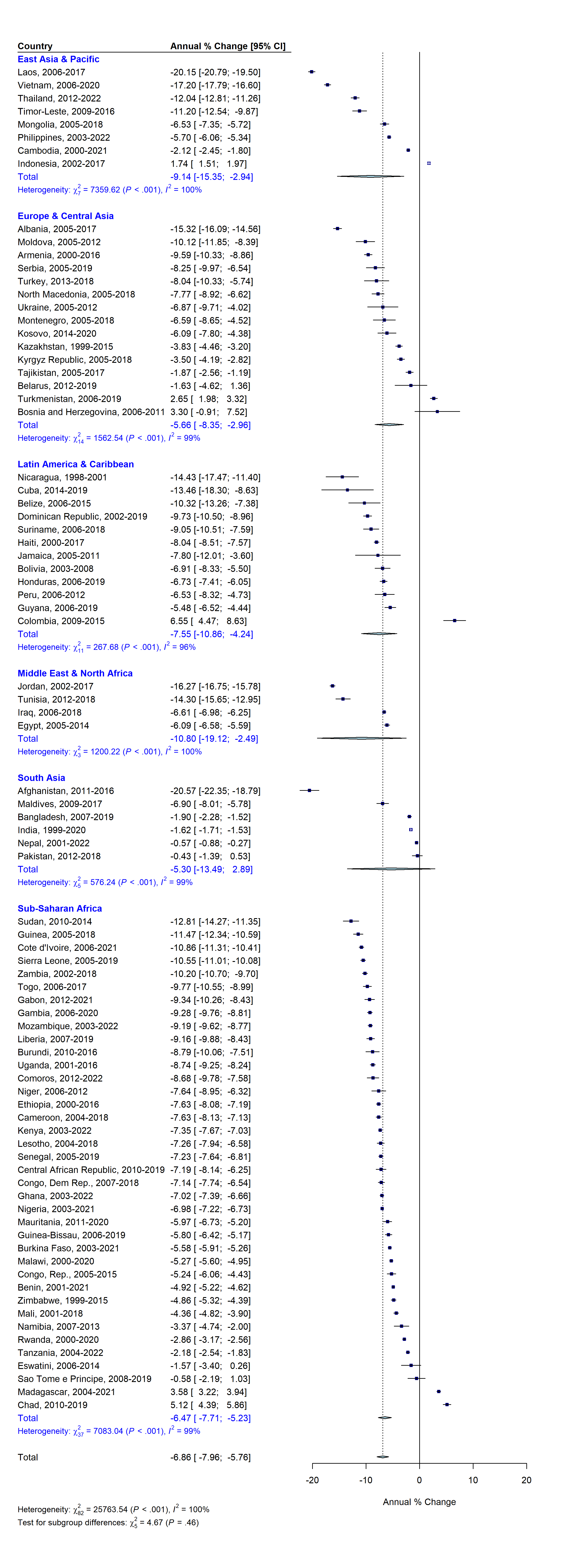


**Figure S3. Global, regional, and country-level annual percentage-point changes in permissive IPV attitudes, men 25 years and under across 57 countries**


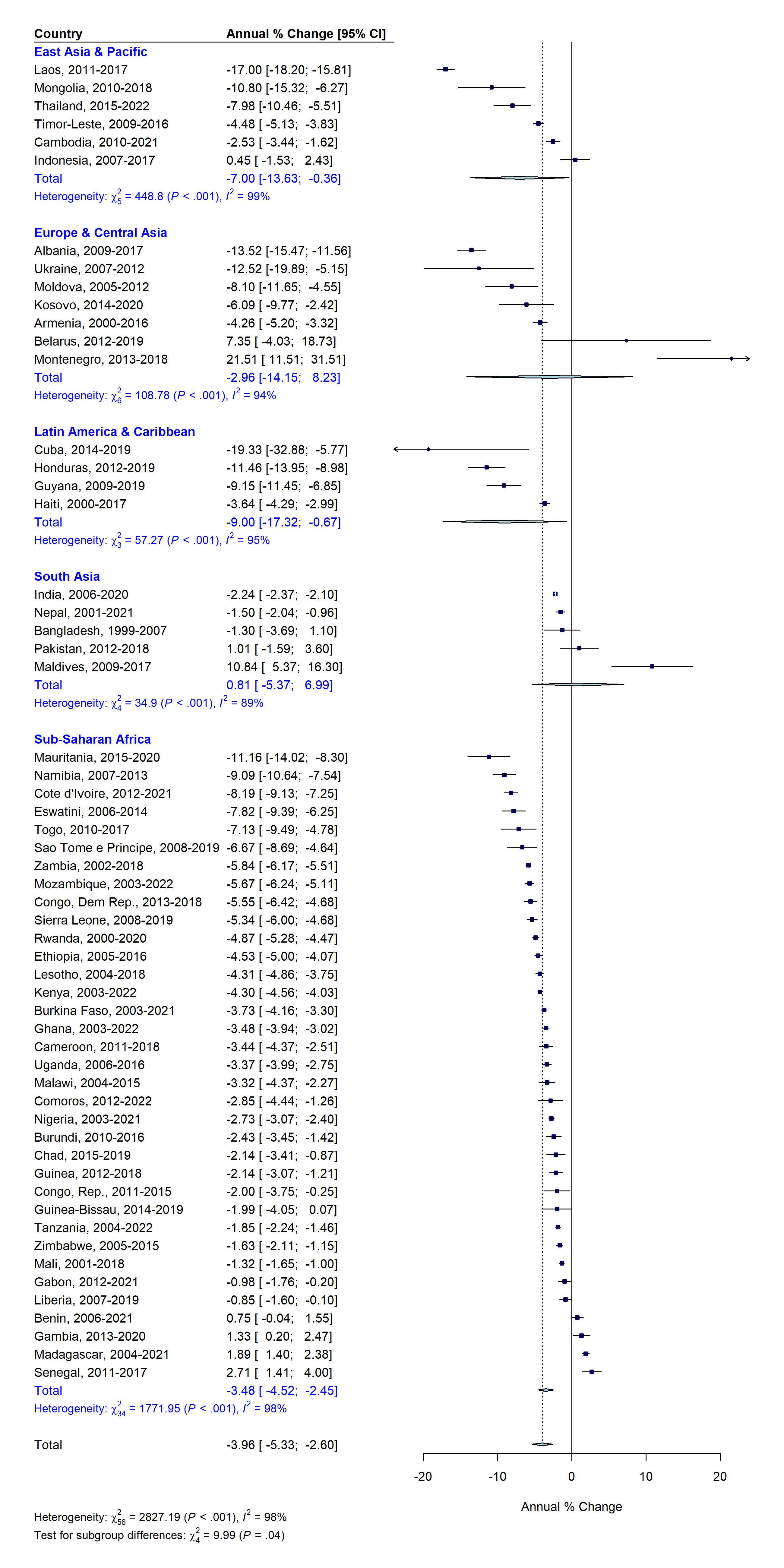


**Figure S4. Global, regional, and country-level annual percentage-point changes in permissive IPV attitudes, men over 25 years across 57 countries**


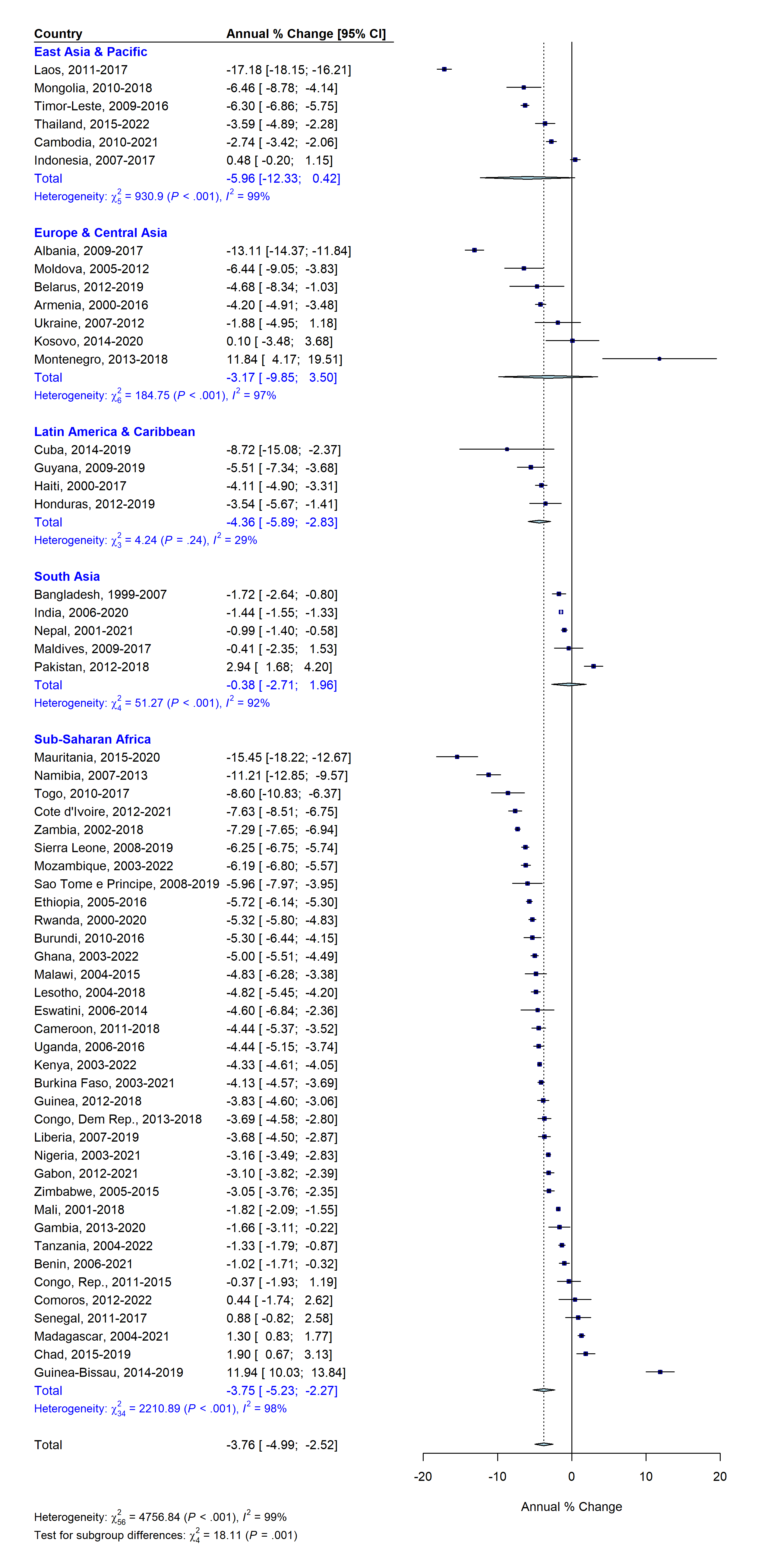

Supplement: Supplementary Materials [file mmc1.docx]
